# Physical function as a marker to assess the effects of occupational long-term pesticide exposure

**DOI:** 10.1371/journal.pone.0300980

**Published:** 2024-05-10

**Authors:** Talita Regina Coelho, Hugo M. Pereira, Ana Tereza Bittencourt Guimarães

**Affiliations:** 1 Laboratory of Biological Investigations, Graduate Program in Biosciences and Health, State University of West Paraná (Unioeste), R. Universitária, Cascavel, PR, Brazil; 2 Department of Health Promotion and Surveillance Federal University of Latin American Integration (Unila), Avenida Tarquínio Joslin dos Santos, Foz do Iguaçu, PR, Brazil; 3 Department of Health and Exercise Science, University of Oklahoma, Norman, OK, United States of America; Federal University of Rio Grande do Sul: Universidade Federal do Rio Grande do Sul, BRAZIL

## Abstract

In this cross-sectional study, we determined the relative impact of long-term occupational exposure to pesticides on physical performance and perception of tiredness. Experimental data was collected in locus from agricultural communities and included surveys to assess the duration of exposure to pesticides, social status, habitual physical activity levels, presence of common mental disorders (CMD), and self-reported tiredness. Plasmatic cholinesterase (PChE), body composition and traditional functional performance tests (Handgrip strength–HGS; Time up and go–TUG; and Sit-to-stand—STS) were obtained. From the 127 individuals tested, cluster analysis yielded 80 individuals divided in Direct Exposed (*n* = 37) and Indirect Exposed (*n* = 43); Tired (*n* = 16), and Not Tired (*n* = 64). PChE values were within the reference values (5209.64–13943.53 U/L). Pesticide exposure had no influence on PChE levels, CMD or fatigue (*p* > 0.05), while Self-reported tiredness had (*p* < 0.05). Principal Component Analyses showed that HGS; STS and TUG (i.e., physical performance variables) are negatively influenced by two independent factors: pesticide exposure and self-reported tiredness. We conclude that chronic pesticide exposure and tiredness can negatively impact physical performance, independently, without clinically significant changes in PChE levels that is a biomarker used to track pesticide intoxication. Functional physical tests can be a useful tool to identify chronic pesticide exposure, and help with the limitations of commonly used parameters (i.e. PChE and CMD). Self-reported tiredness is a confounding variable.

## Introduction

Pesticides play a major role in agricultural activities worldwide and are used to kill, control, or repel pests. Long-term low-level exposure to pesticides can potentially lead to a scenario of chronic intoxication that is difficult to diagnose in humans. To our knowledge, there is a paucity of experimental studies directly evaluating the influence of chronic exposure to pesticides, at levels believed to be safe, on the physical performance of upper and lower extremity muscles. Adequate physical capacity to meet job expectations relies on the integrity of the neuromuscular system [1], and although basic science reports suggest that long-term pesticide exposure can negatively impact multiple mechanisms in the neuromuscular system [2–6], the effects of low-level long-term occupational pesticide exposure on physical performance in men and women are not fully understood.

In the general population, self-reported tiredness impacts 7% to 45% of adults [7], and in one-third of the cases, the cause for this symptom is unknown [8]. Self-reported tiredness is particularly present in middle-aged individuals, thus representing a large proportion of the working class [9]. Additionally, self-reported tiredness can be predictive of other health complications and increase the mortality odds [10]. In individuals not exposed to pesticides, self-reported tiredness was shown to be associated with poor functional performance for both the upper and lower extremity muscles [9,11], which in turn, can negatively impact quality of life [12], increase the risk of depression [13] and mortality [14]. It is not known, however the impact of chronic exposure to pesticides on self-reported tiredness and its association with physical function. Thus, the goal of this study was to determine the association between long-term occupational exposure to pesticides, at levels believed to be safe, and perception of tiredness, as well as metrics of physical function obtained from tests widely used by clinicians to quantify physical performance of the upper and lower extremity muscles. Our hypothesis is that chronic low-level exposure to pesticides will negatively impact physical function and metrics of self-reported tiredness in men and women.

## Materials and methods

### Study sample

This cross-sectional study was possible by establishing partnerships with local communities and organizations that allowed data collection in a small city in the south of Brazil, where the economy is primarily based on agricultural activity. The city is known for its grain production, which primarily involves soybeans, corn, and wheat. Organophosphates are the most commonly commercialized pesticides [15]. We recruited 127 individuals (age range from 19 to 90 years old) from June 14^th^ to August 28^th^, 2021. This sample size was sufficient to produce power of 80% with effect size of 0.3, alpha of 5% for an F-test calculated a priori. Individuals were first invited to participate via phone call. We randomly contacted those individuals previously registered in the Brazilian public health system database, the information was provided by the local department of health. Different urban and rural areas of the city were targeted to prevent sampling bias. After the initial phone interview, investigators visited each participant to explain the study details. Exclusion criteria was presence of a pacemaker, pregnancy; flu-like symptoms; or presence of orthopedic conditions preventing adequate performance of functional tests.

During data collection two questions were used to determine the study groups. Pesticide exposure was determined by the question: “Do you use/apply pesticides?”. Those who answered “yes”, mentioning constantly using pesticide for their work-related activities were considered Direct Exposed and were placed in the “Direct” group. While those who answered “no”, not mentioning purposely manipulating pesticides were considered Indirect Exposed and were placed in the “Indirect” group. “Indirect exposure” was preferred here instead of “not exposed” as some level of unintended exposure is frequently present, especially considering that the city where the study was conducted is surrounded by farms and that pesticide commercialization is high in the region.

Tiredness was determined by self-report. Those who answered “yes” to the question “Do you feel tired all the time?” were placed in the “Tired” group, while those who answered “no” were placed in the Not Tired group. The researchers conducting the field test sessions had no knowledge of the participants groups.

### Measures

The study was conducted in accordance with the Declaration of Helsinki, and approved by the National Ethics Committee (40815120.8.0000.0107; 06/03/2021). Each participant signed a written informed consent. Surveys were used to investigate duration of exposure to pesticides and potential symptoms, level of education as well as social status. This questionnaire was carefully constructed in conjunction with health professionals of the local public health system. The experimental test session was conducted at a community center and the following were obtained:

*Habitual physical activity*: The short form of the International Physical Activity Questionnaire (IPAQ) culturally adapted to the local language classified the levels of habitual physical activity according to previously established norms [16].

*Plasmatic Cholinesterase (PChE)*: This is a biomarker used by health authorities to assess pesticide intoxication [17]. Blood serum was processed by an Automated Kinetic Method with Attellica Technology from Siemens ®. For interpretation, the following normative reference values were used: for men, equivalent to 4,620.0 to 11,500.0 U/L, and for women, 3,930.0 to 10,800.0 U/L. Sample was obtained at rest and before participants perform the functional tests.

*Body Mass Index (BMI)*: Visceral fat, percentage of body fat and skeletal muscle were obtained using an Omron HBF-514 Full Body Bioimpedance Digital Scale. Test procedures followed international guidelines and results were classified by age and sex according to normative values [18].

*Handgrip strength (HGS)*: Participants were seated in a chair, elbow flexed at 90°, forearms supported and wrist in a neutral position. Three measures of the dominant hand were taken, using a Saehan hydraulic dynamometer, with 45 seconds of rest between trials and the maximal value was recorded [19].

*Sit-to-stand (STS)*: Time was recorded for the participant to rise to a full stand and immediately return to the initial seated position five times. The chair had no armrest, seat height of 45 cm and back straight. Individuals had arms crossed against the chest. A familiarization was conducted to prevent learning effect [20].

*Time up and go (TUG)*: Time was quantified for individuals to stand up out of the chair, walk 3 meters, turn around, walk back to the chair, and sit down completely [21]. Test was performed after participants being familiarization with the procedures.

*Mental health and tiredness*: The Self-Reported Questionnaire 20-item (SRQ 20) culturally adapted to the local language was used. This instrument is traditionally used by health authorities to assess chronic intoxications by pesticides. Scores greater than 7 is indicative of risk of common mental disorders (CMD) [22]. Additionally, the question “Do you feel tired all the time?” was used to assess self-reported tiredness.

### Statistical analyses

Given the sample’s high heterogeneity due to a wide age range, a Hierarchical Cluster Analysis was conducted. The primary analysis was the Jaccard similarity index, followed by the Unweighted pair-group average (UPGMA) agglomeration method. Participants with similar age, BMI, and exposure to pesticides were grouped. All qualitative variables were showed with absolute and relative (%) frequencies. Pearson Chi-square tests were used to investigate differences in frequency in each isolated factor ("Pesticide exposure" and "Tiredness"). In cases of statistical significance (p<0.05) the Adjusted Residues post-hoc test was subsequently used.

To account for the potential influence of age and sex differences in physical performance, Z-scores were calculated for HGS, STS and TUG using reference values [20,21]. These data were in agreement with the assumptions of normality and homoscedasticity and therefore, were analyzed considering the factors "Pesticide exposure" and "Tiredness" using Two-way-ANOVA and Tukey-HSD post-hoc test, with a significance of 5% (p < 0.05). Subsequently, the matrices of variables were submitted to multivariate principal component analysis (PCA). The factorial loads of the first two principal components were analyzed considering the factors, also using the same parametric tests.

Finally, the factorial loads obtained by the PCA, were related to the Plasma Cholinesterase values by a linear regression model, to illustrate a possible cause-and-effect relationship. At this stage, the response variables (y) were the factorial loads, and the predictor variables (x) were the values of PChE in exposed and non-exposed, tired, and non-tired individuals.

All analyzes were performed using ‘ggplot2’, ‘Rmisc’, ‘ExpDes.pt’, ‘ggfortify’, ‘dplyr’, ‘vegan’, ‘factoextra’ and ‘psych’ packages of R software (R Core Team, 2020).

## Results

Cluster analysis yielded 80 individuals with a BMI above 24.9 kg/m^2^ and aged between 19 and 59 years old [The number of individuals excluded was: older individuals = 32, low BMI values = 14 and flu-like symptoms = 1]. The sample was divided into 2 factors: Pesticide exposure, with two levels, Direct exposed (Direct)–those who during the interview process declared manipulating pesticides at work, Indirect Exposed (Indirect)–those who did not, and self-reported tiredness, also with two levels, Tired—individuals who answered yes to the SRQ20 survey question, and Not Tired—those who answered no.

### Sample characteristics

Most individuals in the Direct group were men with a high school degree, living in rural areas of the community, aged between 50 and 59 years old, and exposed to pesticides for more than 10 years. The Indirect group had a greater proportion of women, aged between 40 and 49 years old, with a college degree who lived in areas that are relatively more urbanized but within the same geographical location. There were no differences between these groups regarding self-reported fatigue, muscle weakness, muscle pain, risk of CMD, physical activity, and body composition (Table 1). As for the SRQ 20 question “Do you feel tired all the time?” a similar proportion of people answered yes in the Direct (18.9%) and the Indirect (20.9%) group (*p* = 0.64) without interaction between sex and exposure (*p* = 0.258).

**Table 1:** Group characteristics.

	Pesticide Exposure			Tired		
Characteristics	Direct, n (%) *n* = 37	Indirect, n (%) *n* = 43	*p*-value^a^	Tired, n (%) *n* = 16	Not Tired, n (%) *n* = 64	*p*-value^a^
**Sex**			**0.003**			**0.002**
Women	10 (27.0)	26 (60.5)*		13 (81.3)*	23 (35.9)	
Men	27 (73.0)*	17 (39.5)		3 (18.8)	41 (64.1)*	
**Level of education**			**0.011**			0.518
Primary school	19 (51.4)*	9 (20.9)		7 (43.8)	21 (32.8)	
High School	10 (27.0)	14 (32.6)		3 (18.8)	21 (32.8)	
College Degree	8 (21.6)	20 (46.5)*		6 (37.5)	22 (34.4)	
**Age**			0.016			0.375
19–29	4 (10.8)	10 (23.3)		5 (31.3)	9 (14.1)	
30–39	7 (18.9)	4 (9.3)		1 (6.3)	10 (15.6)	
40–49	6 (16.2)	17 (39.5)*		4 (25.0)	19 (29.7)	
50–59	20 (54.1)*	12 (27.9)		6 (37.5)	26 (40.6)	
**Housing Area**			**0.000**			0.733
Rural	23 (62.2)*	10 (23.3)		6 (37.5)	27 (42.2)	
Urban	14 (37.8)	33 (76.7)*		10 (62.5)	36 (57.8)	
**Symptoms**						
Fatigue	11 (27.0)	13 (30.2)	0.752	11 (68.8)*	12 (18.8)	**< 0.0001**
Muscle weakness	12 (32.4)	11 (25.6)	0.595	11 (68.7)*	12 (18,7)	**0.000**
Muscle Pain	21 (56.8)	30 (69.8)	0.227	14 (87.5)*	37 (57.8)	**0.027**
**Duration of Pesticide Exposure**			**< 0.0001**			0.319
Unexposed	0 (0.0)	42 (97.7)*		9 (56.3)	33 (51.6)	
Less than 1 year	3 (8.1)	1 (2.3)		0 (0.0)	4 (6.3)	
1 to 5 years	3 (8.1)*	0 (0.0)		1 (6.3)	2 (3.1)	
5 to 10 years	4 (10.8)*	0 (0.0)		1 (6.3)	3 (4.7)	
More than 10 years	27 (73.0)*	0 (0.0)		5 (31.3)	22 (34.4)	
**SRQ 20**			0.481			**< 0.0001**
Mental Health risk	11 (29.7)	16 (37.2)		15 (93.7)*	12 (18.7)	
**IPAQ**			0.249			0.177
Very Active	12 (33.3)	7 (16.7)		3 (20.0)	16 (25.4)	
Active	15 (41.7)	16 (38.1)		5 (33.3)	26 (41.3)	
Irregularly active A	4 (11.1)	10 (23.8)		5 (33.3)	9 (14.3)	
Irregularly active B	1 (2.8)	4 (9.5)		2 (13.3)	3 (4.8)	
Sedentary	4 (11.1)	5 (11.9)		0 (0.0)	9 (14.3)	
**% Muscle**			0.506			0.584
Low	27(73.0)	34 (79.1)		14 (87.5)	47 (73,4)	
Regular	9 (24.3)	9 (20.9)		2 (12.5)	16 (25.0)	
High	1 (2.7)	0 (0)		0 (0.0)	1 (1.6)	
**% Body Fat**			0.227			0.576
Regular	2 (5.4)	1 (2.3)		0 (0.0)	3 (4.7)	
High	9 (24.3)	5 (11.6)		2 (12.5)	12 (18.8)	
Very High	26 (70.3)	37 (86.0)		14 (87.5)	49 (76.6)	
**% Visceral Fat**			0.177			0.351
Regular	9 (24.3)	19 (44.2)		8 (50.0)	20 (31.3)	
High	17 (45.9)	15 (34.9)		6 (37.5)	26 (40.6)	
Very High	11 (29.7)	9 (20.9)		2 (12.5)	18 (28.1)	

Data is reported in absolute (n) and relative (%) values of direct and indirect exposed to pesticides (Direct and Indirect, respectively), tired all the time and not tired (Tired and Not Tired, respectively), as well as interaction between pesticide exposure and self-reported tiredness. ^a^*P* values in bold indicates statistical significance for chi-square (*p*<0.05). * indicates statistical significant difference between/among categories.

A greater proportion of women reported being tired all the time than men (*p* = 0.002), regardless of duration of pesticide exposure (*p* = 0.319). Table 1 also shows that risk of CMD, fatigue, muscle weakness, and muscle pain had greater prevalence in the Tired group (all *p* <0.05). Both groups had similar education, housing, habitual physical activity levels, and body composition (all *p* > 0.05).

### Physical performance

Pesticide exposure or self-reported tiredness had no influence on the results of the TUG (all *p* > 0.05). However, HGS and STS z-scores were lower in individuals in the Tired group compared with the Not Tired group (*p* < 0.05) without influence of pesticides exposure (*p* > 0.05) or interaction (*p* > 0.05; Table 2). We used z-scores in these comparisons to minimize the influence of sex and age on the results.

**Table 2:** Handgrip strength, time up and go, and sit-to-stand. Data is mean and standard deviation of the Z-score.

	Pesticide Exposure			Tired			Interaction				
	Direct, mean (SD) *n* = 37	Indirect, mean (SD) *n* = 43	*p*-value^a^	Tired, mean (SD) *n* = 16	Not Tired, mean (SD) *n* = 64	*p*-value^a^	Direct and Tired, mean (SD) *n* = 7	Direct and Not Tired, mean (SD) *n* = 30	Indirect and Tired, mean (SD) *n* = 9	Indirect and Not Tired, mean (SD) *n* = 34	*p*-value^a^
**Handgrip Strength**	-0.967 (0.965)	-0.583 (1.177)	0.097	-1.508 (1.166)	-0.574 (1.001)	**0.001**	-1.457 (1.249)	-0.853 (0.874)	-1.547 (1.172)	-0.328 (1.054)	0.287
**Time Up and Go**	1.616 (1.609)	1.267 (1.508)	0.324	1.638 (1.441)	1.376 (1.589)	0.536	1.795 (1.065)	1.574 (1.723)	1.515 (1.734)	1.201 (1.464)	0.916
**Sit-to-stand 5x**	1.411 (1.072)	1.400 (0.948)	0.958	1.971 (1.288)	1.264 (0.871)	**0.011**	2.158 (1.238)	1.237 (0.971)	1.825 (1.380)	1.287 (0.787)	0.488

Value of 1 represents the result is closest to reference values relative to sex and age. For the handgrip strength, negative values represent poorer outcomes. As for Time up and go and Sit-to-stand 5x, positive values indicate poor results.

SD: Standard Deviation.

^a^*P* values in bold indicates statistical significance for Two-way ANOVA (p<0.05).

### PChE analysis

**Fig 1:**
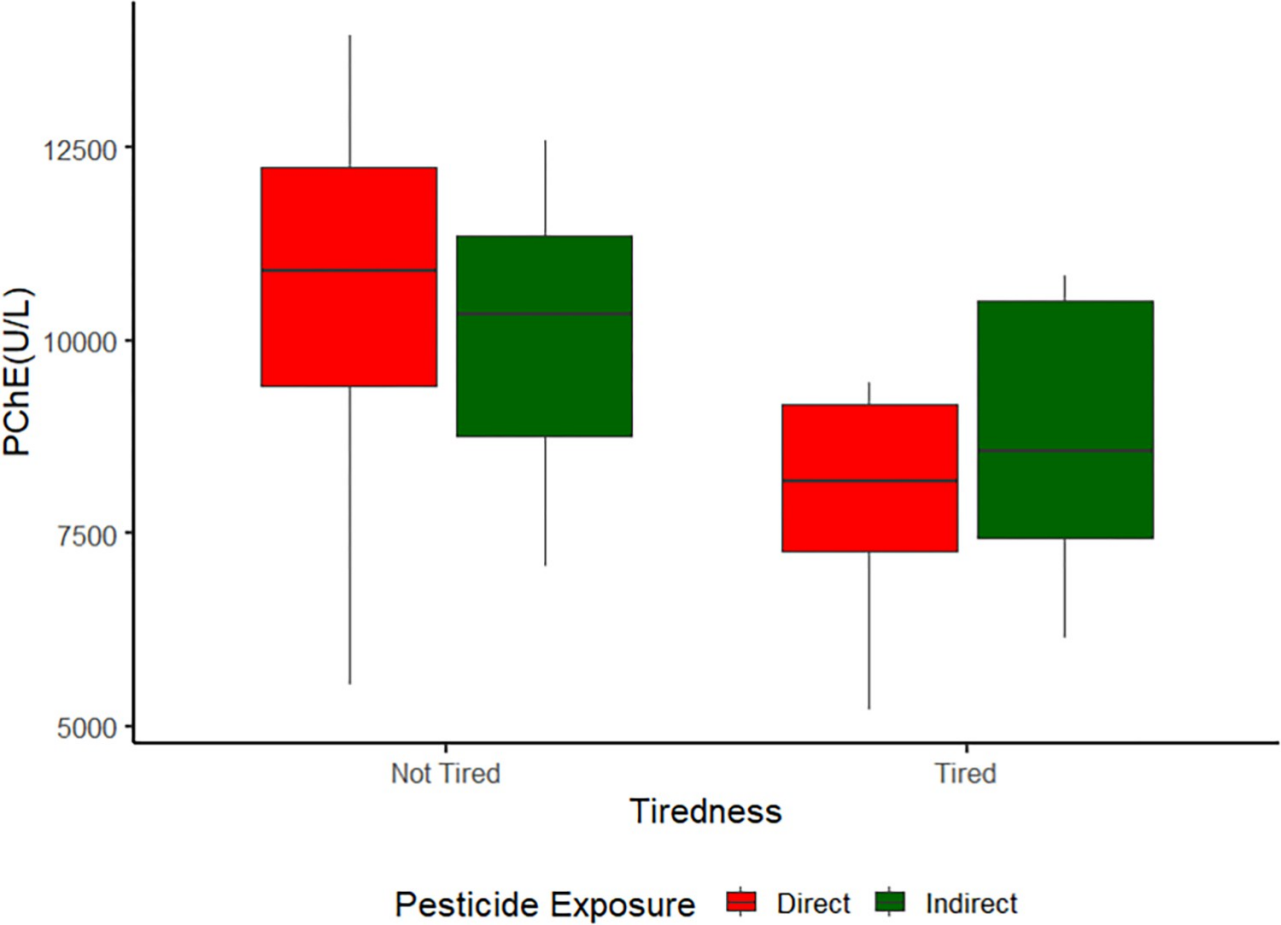
Plasmatic Cholinesterase (PChE). Boxplot of Plasmatic Cholinesterase activity between groups Direct and Indirect; Tired and Not Tired.

All individuals had values within the reference range PChE (5209.64–13943.53 U/L). Tired group had lower values for PChE than those in the Not Tired group (*p* = 0.001). No influence of pesticide exposure (*p* = 0.152) or interaction were observed (*p* = 0.240; Fig 1).

### Integrative assessment

**Fig 2:**
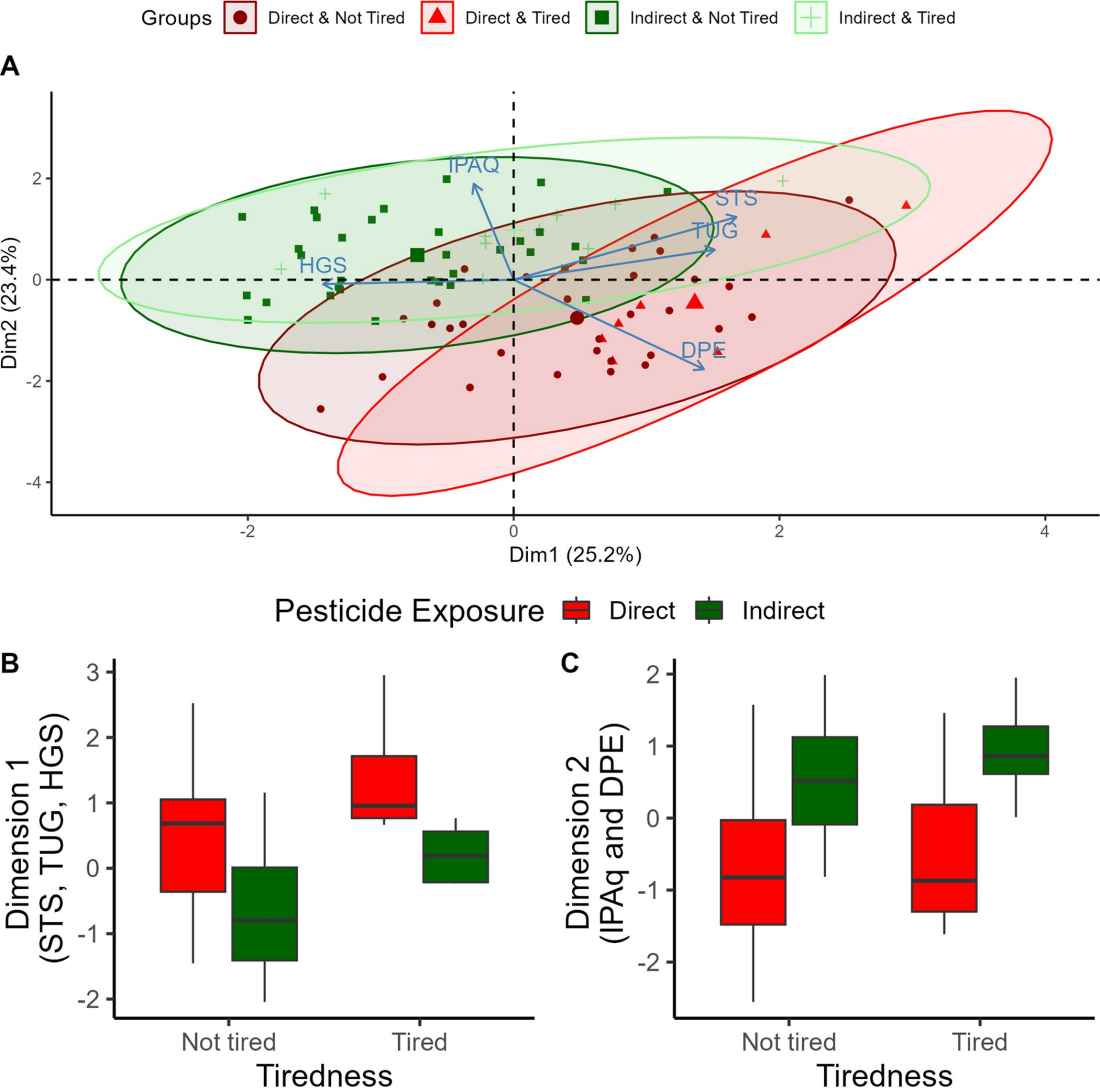
Diagram of integrative assessment through Principal Component Analysis (PCA). A) PCA ordering diagram, indicating Dimension 1 –Functional Performance (Sit to Stand–STS; Time Up and Go–TUG; Handgrip Strength HGS) and Dimension 2 –Physical Activity (IPAQ) and Duration of Pesticide Exposure (DPE) for the interaction between Perception of Tired and Pesticide Exposure. B) Boxplot of Dimension 1 –Functional Performance between groups Direct and Indirect; Tired and Not Tired. Positive factorial loads indicate worse performance. C) Boxplot of Dimension 2 –Physical Activity and Duration of Exposure between groups Direct and Indirect; Tired and Not Tired. Positive factorial loads represent people who are more sedentary and have a shorter DPE.

PCA was conducted to identify combined factors characterizing groups of individuals. Two main dimensions were detected, with an accumulated explicability of 48.61% (Fig 2A). The first dimension highlighted the influence of STS, TUG and HGS representing 30.2%, 24.6% and 22.1% of the dimension respectively (Eigenvalue = 1.26; Variance = 25.23%). Physical activity (i.e., IPAQ results) and duration of pesticide exposure (DPE) had the biggest contribution for the second dimension, each with 41.8%, and 36.3%, respectively (Eigenvalue = 1.17; Variance = 23.37%).

In the first dimension, functional performance, positive values indicate higher times to STS and TUG and lower HGS. When comparing the factorial loads for this dimension, main effect of direct exposure (*F*_1,76_ = 35.126; *p* < 0.001) and presence of self-reported tiredness was found (*F*_1,76_ = 12.651; *p* = 0.00065), with no interaction (*F*_1,76_ = 0.002; *p* = 0.96325). Thus, people in the Direct group had worse functional performance when compared to the Indirect group. Worse functional performance was also present in the Tired group, compared to the Not Tired group (Fig 2B).

In the second dimension, that includes physical activity and DPE, negative factorial loads represent people who are more active and have a longer DPE. Those in the Direct group had a longer DPE and were more physically active than those in the Indirect group (*F*_1,76_ = 43.795; *p* = <0.0001). Tiredness (*F*_1,76_ = 1.909; *p* = 0.17112) or the interaction between pesticide exposure and tiredness had no influence on the second dimension (*F*_1,76_ = 0.032; *p* = 0.85860; Fig 2C).

### PChE vs. Physical performance

We further investigated the influence of PChE in each PCA dimension and the factors Pesticide Exposure (i.e., Direct vs Indirect) and presence of self-reported tiredness (Tired and Not Tired). No influence of PChE was found in the first (*t* = -1.307; *p* = 0.195) or second dimensions (*t* = -1.105; *p* = 0.273).

## Discussion

Our study investigated the effects of chronic exposure to pesticides on metrics of physical performance typically used by clinicians (TUG, STS, HGS) as well as tiredness, which is a self-reported metric strongly associated with overall quality of life and mortality. Our findings are novel and indicate that individuals chronically exposed to low levels of pesticides in the work environment have worse physical function of upper and lower extremity muscles compared to the individuals not directly exposed to pesticides (Fig 2B) without disrupting PChE levels. Additionally, individuals reporting being tired all the time had lower physical performance and this is independent of pesticide exposure (i.e., PCA findings). These findings were observed after accounting for potential age and sex differences in motor performance. Combined, these observations suggest that physical function can potentially be used as an alternative marker to help with the intrinsic limitations of PChE on detecting chronic intoxications by pesticides. Additionally, self-report of tiredness act as a confounding variable as it was associated with worst values of physical function regardless of pesticide exposure.

Although the physiological mechanisms explaining our physical performance findings are yet to be fully elucidated, previous observations showed that pesticides intoxication, can affect several vital mechanisms in the neuromuscular system and thus affect physical performance. For instance, organophosphates and carbamates, widely used in agriculture, can inhibit acetylcholinesterase, causing a blockage of the neuromuscular junction by accumulation of acetylcholine, potentially disturbing the ability of a muscle to contract [4]. They also can raise mitochondrial Ca^++^ levels impairing the mitochondrial complex and inducing oxidative stress, which may lead to neurodegeneration [23]. Electromyographic studies in humans also show that long-term low-level pesticide exposure was associated with the presence of peripheral neuropathy [5,24,25].

Although PChE is traditionally used as a marker to demonstrate intoxication by organophosphates and carbamates exposure [26], there is substantial evidence that this inhibition is unlikely to be the only mechanism explaining the variety of symptoms reported [23]. Results from the current study suggest that long-term pesticide exposure has no impact on PChE concentrations. However, even with PChE activity within reference values, long-term pesticide exposure can negatively impact motor performance during activities that simulate work-related tasks and activities of daily living. Our observations provide opportunities for future studies investigating alternatives to the reliance on PChE levels when diagnosing occupational long term pesticide exposure, which can be of tremendous importance for clinicians. Additionally, it is crucial to account for the role of self-reported tiredness as a confounding variable, as it can affect PChE activity and physical performance without any association with pesticide exposure.

Detecting chronic intoxication by pesticides is challenging. Using other assessment tools, such as perceived metrics, could facilitate the chronic intoxication identification. Our data suggest that self-reported tiredness used in isolation was not able to discriminate individuals chronically exposed to pesticide. Specifically, and contrary to others [27–30], we did not find difference in the proportion of individuals reporting tiredness between the Direct and Indirect groups. Additionally, in our study, unlike others [2,3,28], there were no association between pesticide exposure and self-report of fatigue, muscle weakness or pain. However, tiredness was associated with lower physical performance of hand muscles and lower extremities (i.e. PCA findings). Our results are in agreement with previous reports showing that self-reported tiredness was negatively associated with objective metrics of physical performance in middle-aged and older individuals not exposed to pesticides (>65 years old) [9,11,31]. Considering that self-reported tiredness is a predictor of functional limitations and disabilities [32,33], combined these observations are relevant for the workforce as approximately 21.5% of our sample, which is composed primarily of middle-aged working individuals, as well as 37.9% of the workforce in another country frequently report being tired [7].

We observed a higher prevalence of self-reported tiredness in women than in men (4:1). This is in accordance with some [7,34] but not others [35] that investigated individuals not exposed to pesticides. Determinants of sex differences in self-reported tiredness are yet to be fully understood and potentially involve sex differences in the neuromuscular physiology involved on the physical demands to adequately accomplish daily and work-related tasks [36], as well as sex differences in other physiological function (e.g. contraception, menstruation and pregnancy) and social factors (e.g. combine employment with caring for young children) [37].

CMD risk is one of the parameters proposed by health authorities to assess pesticide intoxications. Pesticide exposure has the potential to disturb neurochemistry function and, hence, predispose individuals to psychological disorders, but data on chronic low-level exposure is limited and unclear [38]. In our study, pesticide exposure was not associated with risk of CMD which is similar to a previous observation from Bazo-Alvarez [39], reporting that higher risk of mental disorders was associated with heavy workloads, but not with pesticide exposure. Nevertheless, our data showed that tiredness is associated with an elevated risk for CMD, as previously shown [9,40]. These results again suggest that tiredness can bias the results of chronic intoxication by pesticides and CMD is not a reliable parameter to identify individuals chronically exposed.

### Limitations

An intrinsic limitation of this cross-sectional study is determining the cause-effect relationship between these variables. To be able to adequately investigate individuals who are constantly working with pesticides, we intentionally sampled individuals from an area where the economy is primarily based on agricultural activity. The individuals not directly exposed to pesticides were sampled from the same geographical area for adequate paired comparison. However, it is worth to mention the per capita pesticide consumption was remarkably high in the regional area, which could potentially lead to chronic environmental pesticide exposure in individuals not directly exposed to pesticides. Future studies should focus on comparing communities with the same occupation but different agricultural approaches (e.g., conventional vs. organic).

## Conclusion

This study suggests that chronic occupational pesticide exposure may negatively impact physical performance without clinically important alterations in PChE activity. Additionally, self-reported tiredness paralleled muscle pain, upper extremity muscle weakness, high risk of CMD and poor performance in functional tests of the lower extremities that resemble daily activities. There are tremendous opportunities for future studies assessing the association between physical performance, long-term pesticide exposure and tiredness. These findings have potential implications to clinicians and future public health policies as they can provide evidence that commonly used parameters (i.e PChE; CMD) to assess intoxications by pesticides have limitations that can be supplemented by using traditional clinical tools used to assess physical function.
